# Comparison of fluid dynamics changes due to physical activity in 3D printed patient specific coronary phantoms with the Windkessel equivalent model of coronary flow

**DOI:** 10.1186/s41205-022-00138-8

**Published:** 2022-04-07

**Authors:** Kelsey N. Sommer, Mohammad Mahdi Shiraz Bhurwani, Vijay Iyer, Ciprian N. Ionita

**Affiliations:** 1 Department of Biomedical Engineering, University at Buffalo, Buffalo, NY USA; 2 Canon Stroke and Vascular Research Center, University at Buffalo, Buffalo, NY USA; 3 QAS.AI Incorporated, Buffalo, NY 14203 USA; 4 University at Buffalo Cardiology, University at Buffalo Jacobs School of Medicine, Buffalo, NY USA

**Keywords:** 3D Printing, Coronary Artery Disease, Coronary CTA, Windkessel model

## Abstract

**Background:**

3D printing (3DP) used to replicate the geometry of normal and abnormal vascular pathologies has been demonstrated in many publications; however, reproduction of hemodynamic changes due to physical activities, such as rest versus moderate exercise, need to be investigated. We developed a new design for patient specific coronary phantoms, which allow adjustable physiological variables such as coronary distal resistance and coronary compliance in patients with coronary artery disease. The new design was tested in precise benchtop experiments and compared with a theoretical Windkessel electrical circuit equivalent, that models coronary flow and pressure using arterial resistance and compliance.

**Methods:**

Five phantoms from patients who underwent clinically indicated elective invasive coronary angiography were built from CCTA scans using multi-material 3D printing. Each phantom was used in a controlled flow system where patient specific flow conditions were simulated by a programmable cardiac pump. To simulate the arteriole and capillary beds flow resistance and the compliance for various physical activities, we designed a three-chamber outlet system which controls the outflow dynamics of each coronary tree. Benchtop pressure measurements were recorded using sensors embedded in each of the main coronary arteries. Using the Windkessel model, patient specific flow equivalent electrical circuit models were designed for each coronary tree branch, and flow in each artery was determined for known inflow conditions. Local flow resistances were calculated through Poiseuille’s Law derived from the radii and lengths of the coronary arteries using CT angiography based multi-planar reconstructions. The coronary stenosis flow rates from the benchtop and the electrical models were compared to the localized flow rates calculated from invasive pressure measurements recorded in the angio-suites.

**Results:**

The average Pearson correlations of the localized flow rates at the location of the stenosis between each of the models (Benchtop/Electrical, Benchtop/Angio, Electrical/Angio) are 0.970, 0.981, and 0.958 respectively.

**Conclusions:**

3D printed coronary phantoms can be used to replicate the human arterial anatomy as well as blood flow conditions. It displays high levels of correlation when compared to hemodynamics calculated in electrically-equivalent coronary Windkessel models as well as invasive angio-suite pressure measurements.

## Background

3D printing (3DP) offers the ability to engineer unique three-dimensional objects in varying materials and boasts in its ability to develop prototypes or functional models at a rapid pace while maintaining the complexity of the geometry. While traditional manufacturing methods are still generally cheaper for mass production, 3D printing is ideal for creating one-of-a-kind customizable items. Recent literature has highlighted 3DP being potentially used in personalized medical approaches, to date including the engineering of bandages, surgical guides, prosthetics, etc. [1–3]. In addition, 3DP has opened new opportunities for the medical community through the creation of geometrically accurate patient specific anatomy for treatment planning, resident training, device testing and physiological benchtop simulations [4–10].

Vascular 3D printed models in particular pose significant challenges due to complex geometry, inability of the 3D printers to render small arteries and unavailability of materials which mimic accurately the arterial wall mechanical properties that are directly related to the hemodynamics. The compliance of the material especially, is a key component for proper simulation of artery wall compliance which ultimately will affect hemodynamic conditions [11]. In recent work on 3D printing of the coronary arteries, researchers attempted to improve the relevance of hemodynamic simulations by manipulating the compliance of the large arteries using different soft 3D printing materials [12–14]. For coronary artery disease (CAD), in addition to the vessel wall compliance, proper inclusion of the plaque within the vessel lumen while reproducing the mechanical properties, is also essential since it may affect the local and global hemodynamics within the affected coronary tree [15]. Namely, the stenosis within the coronary arteries with CAD has two effects. First it adds epicardial resistance [16, 17] to coronary blood flow leading to possible ischemia, and second, it will change the local compliance of the artery. Hence, proper compliance of the calcification within the coronaries is needed to improve the accuracy of the benchtop simulations.

A third key condition for accurate simulations is the boundary conditions which allow autoregulation of the arterial network and the hemodynamics within the coronary arteries. The distal arterial network is crucial in representing the arterioles and the capillary bed; however, 3DP technology is not capable of printing distal arterioles small and thin enough.

Some of the constraints outlined above may be achieved using recent advances in 3D printing. Compliance of the arterial wall, for example, may be controlled using soft materials such as Flexible photoresins (Stratasys Tango family (Stratasys, Eden Prairie, MN)) and hard photoresins (Stratasys Vero family). In addition, multi-material 3D printing capabilities allows manufacturing of patient specific models which include calcification within the coronary arteries representing varying disease state severities which have different mechanical properties than the arterial wall. Rendering of the capillary bed, however, it is not possible with current 3D printing technologies at this time. Alternative solutions have been proposed by building special constructs to simulate capillary bed flow properties including adjustment of its hemodynamic properties as a function of the physical effort. The solution consists of an adjustable compliance chamber which collects the flow of a given coronary tree and adjustable flow resistances [12].

With the incorporation of both resistance and compliance within the benchtop simulation setup, this allows for more control of the simulation of the coronary system which could lead to simulations with increased accuracy. In this paper we propose to use this benchtop system to validate and refine a theoretical electrically equivalent model by comparing 3D printed benchtop model physiological results at the location of stenosis with the incorporation of both resistance and compliance [18]. We propose to investigate a new design for patient specific coronary phantoms, which allow adjustable physiological variables such as coronary distal resistance and capillary bed compliance in patients with coronary artery disease. The new design was tested in precise benchtop experiments and compared with a theoretical Windkessel electrical circuit equivalent, that models coronary flow and pressure using arterial resistance and compliance. Using this approach, patient specific electrical circuit models simulating the coronary tree branches in series/parallel can be created, and localized flow rates determined at the points of disease. Since in our 3D printed models and simulations we know precisely the geometry, the mechanical properties and the boundary conditions, the benchtop measurements and the theoretical model results should corelate strongly. The ultimate goal is to use such mathematically-driven models, to improve the design of the boundary conditions constructs which simulate the capillary bed effect and to allow a scientifically supported 3D printing material selection before manufacturing such vascular models. This approach would allow physiologically relevant models which could more accurately render arterial mechanical parameters and reproduce blood pressure and flow rates [19].

## Methods

### Study design

The protocol for collection and the data analysis was approved by the Institutional Review Board (IRB) at Gates Vascular Institute. Seventy-five patients underwent clinically indicated elective invasive coronary angiography (ICA) with a first generation 320-detector row CCTA (Aquilion ONE, Canon Medical Systems, Tustin, CA) prior to the procedure. Scan parameters include 0.5 mm slice thickness, automated tube current modulation, 100 kVp, and a reconstructed voxel size of 0.625 × 0.625x0.5 mm. To minimize the segmentation errors, five patient coronary trees with the least blooming artifacts and patient motion were selected for this study. To increase diseased artery samples, the selection criteria included the presence of coronary artery disease in both the Left Anterior Descending (LAD) and the Left Circumflex (LCX) with a recorded I-FFR for this study. Invasive fractional flow reserve (I-FFR) was recorded via pressure wire at two lesion lengths past the distal end of the lesion for both the Left Anterior Descending (LAD) and Left Circumflex (LCX). For this cohort, there were no measurements acquired in the Right Coronary Artery (RCA). An I-FFR value of less than or equal to 0.8 indicated a hemodynamically significant lesion with the need for a stent to be deployed at the location of the stenosis. From these 5 selected cases, 10 I-FFR measurements were taken in the catheter lab and used as the ground truth for this study (LAD and LCX measurement for 5 cases). Seven out of the 10 I-FFR measurements used in this study indicated the need for stent deployment (5 LAD, 2 LCX <  = 0.8 I-FFR) and 3 I-FFR measurements indicated no need for stent deployment (3 LCX > 0.8 I-FFR). The patient CCTA data was imported into Vitrea (Vital Images, Minnetonka, MN) and the cardiac analysis application was used to manually determine the mean radii of the vessel (at the location of the stenosis) as well as the distance (ostium to I-FFR measurement).

### 3D printed model design

**Fig. 1:**
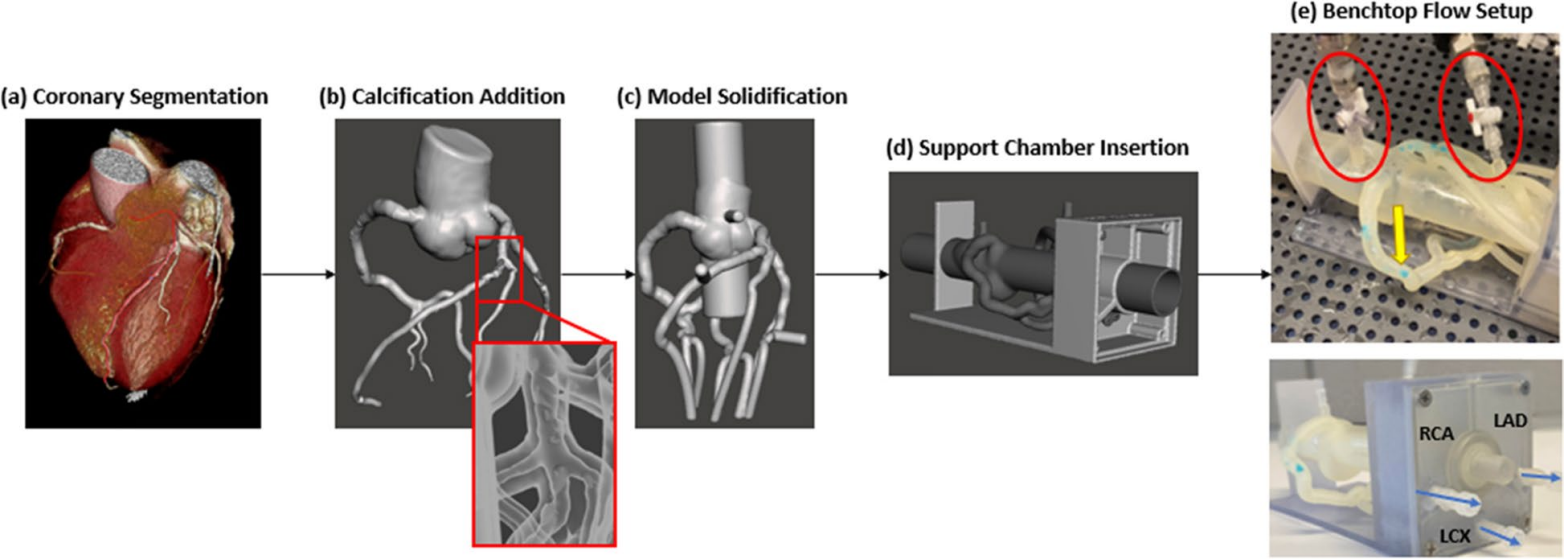
Benchtop Model Development. **a** CCTA scans of the heart tissue and the three main coronary arteries were imported into Vital Images. The cardiac application automatically segmented the aorta and the three main coronary arteries from the rest of the heart tissue. Manual segmentation was additionally implemented using Hounsfield thresholding and contouring methods. **b** The calcification within the coronary arteries was segmented separately from the rest of the heart tissue using Hounsfield thresholding. Both the vasculature and the calcification were imported into Autodesk Meshmixer for sculpting and artifact reduction. **c** A 2 mm wall was generated and a hollow lumen was created to allow for fluidic flow through the solid geometry. **d** A support structure was created in SolidWorks and imported into Autodesk Meshmixer where it was aligned with the vasculature. Each of the three main coronary arteries and their branches were aligned so that they perpendicularly connected to the corresponding chamber. **e** The vasculature was 3D printed, cleaned, and attached to a flow loop. Pressure sensors (red circles) were connected to the aorta and three main coronary arteries for pressure measurement recording. The calcification was printed within the models to properly simulate the disease state (yellow arrow). Catheters of varying lengths and diameters were attached to the chamber outlets (blue arrows) to simulate the distal resistance of the coronary arteries

The methodology to develop these phantoms was described in detail in a previous publication (Sommer et.al) [20] and we will only briefly describe it in this article. The patient CCTA data was used within Vitrea (Vital Images, Minnetonka, MN) cardiac analysis application for automatic segmentation of the aortic root, LAD, LCX, and RCA (Fig. 1a). Manual editing of the vessel centerlines and contours by an expert user was applied to each model to ensure high segmentation accuracy. The calcification was segmented separately from the vasculature using the “organ selection tool” as well as manual contrast thresholding to increase the plaque volume accuracy (Fig. 1b). A stereolithographic (STL) file of the cardiac segmentation and the calcification were created within Vitrea and then separately exported into Autodesk Meshmixer (San Rafael, California) where 3DP models were generated using a method previously described [12]. The segmentation and mesh manipulation process included removing smaller vessel branches, appending ports to the aortic root and three main coronary arteries for future pressure sensor insertion at the locations where I-FFR was measured, and vessel wall design (Fig. 1c). A Stratasys J750 multi-material printer was used (Stratasys, Eden Prairie, MN) to print the vessels, calcification, and a support structure for the model as a single unit all appended together. The models were printed in a soft/elastic material, Agilus (Stratays, Eden Prairie, MN) which best simulates the compliance of the vasculature as seen in previous studies by our group [13, 14]. The compliance assessment was conducted by submerging 3D printed cylinders at 1 mm thickness and 4.5 mm outer diameter in body temperature water within a compliance chamber and by measuring the changes in vessel diameter for various internal pressures within human range. For less than 2 mm thickness walls, two Stratasys 3D printing materials, Tango + and Agilus, displayed compliances within healthy human vessel range which is 0.075 mm^2/mmHg to 0.120 mm^2/mmHg [21]. The calcification was printed in Vero (Stratasys, Eden Prairie, MN), a hard material to match the compliance of the calcification within the vessels.

To preserve the coronary tree geometry, a support structure was created in Solidworks (SolidWorks Corporation, Waltham, MA). The support structure was specifically engineered to doubly act as a three-chambered distal resistance simulator (Fig. 1d). The chamber collects the outflow of the coronary arteries and its daughter branches with each of the three chambers corresponding to each of the three main coronary arteries. In addition to the collection of flow from the three main coronary arteries, the chambers allow an adjustable air cup to control the simulated distal arterial bed compliance, as well as allow adjustable distal flow resistance to simulate at rest and various levels of physical activity coronary flow resistance.

### Benchtop flow setup

Each 3D oriented patient specific model was connected to a flow loop. A programmable pulsatile pump (Compuflow, Shelley Medical Imaging Technologies, Toronto, Ontario, Canada) was used within the flow loop to provide a standard aorta pulsatile flow waveform as described by Kim et. al. [22]. The viscosity of blood was created to be approximately 3.7 cP by mixing a solution of 40% glycerol and 60% water. Pressure sensors were connected to the model at the aorta and at the location along the LAD and LCX where the I-FFR was recorded (Fig. 1e).

Catheters of varying lengths and diameters were connected distal to the support chamber to simulate the coronary vascular beds during activity states of rest, light exercise, and moderate exercise [22]. Since the distal hydraulic resistance is inversely proportional to the level of physical activity, a change in coronary dilation is induced via catheter diameter and length variation. As the physical activity is elevated, the resistance decreases to allow for an increased coronary flow volume to make its way through the arteries. These resistance levels (50,000–300,000 dynes*sec/cm^5^) have been achieved using catheter lengths of 17–112 cm in size 4, 5, and 6 French catheters reported in Sommer, et al. [12].

Pressure was monitored using ports attached to the aorta and the 3 main coronary arteries at the location that the I-FFR was measured. Pressure measurements were taken with flow rates ranging from 250–500 mL/s based on the level of activity induced by the attached catheters. The benchtop FFR was calculated for each of the 5 patient specific models for both the LAD and LCX at states of rest, light exercise and moderate exercise.

### Windkessel electrical equivalent model

**Fig. 2:**
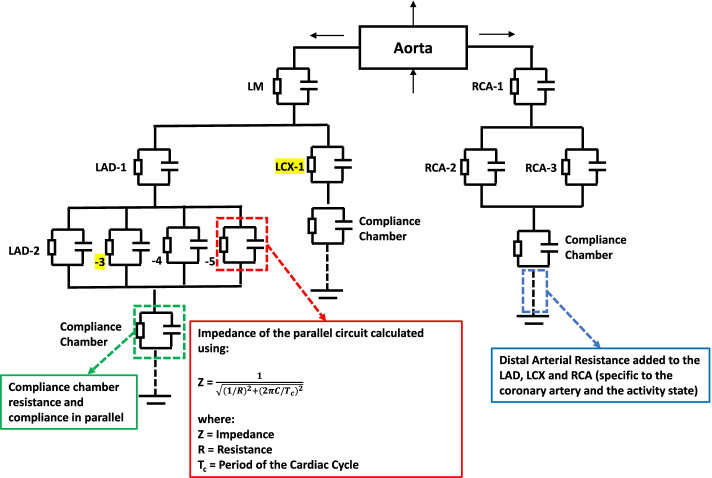
Windkessel Electrical Equivalent Circuit. Using the Windkessel model, patient specific electrical circuit models simulating the coronary tree branches in series/parallel were created, and localized flow rates at the points of stenosis were determined. This schematic displays the Left Anterior Descending (LAD), Left Circumflex (LCX), and Right Coronary Artery (RCA) branches. Highlighted branches indicate that the I-FFR was determined along this branch (distance along the vessel not included in figure)

For every 3D printed model, a Windkessel circuit was designed to account for the aorta, coronary trees, the compliance chamber and the adjustable activity state resistance. In the Windkessel formulation, the simulation domain is decomposed into segments, connected to each other at nodes corresponding to each arterial branching. Each segment is modelled as a deformable tube, representing a blood vessel, whose properties are described by a local resistance and compliance. This theoretical electrically equivalent approach models a schematic diagram created for each patient specific model (Fig. 2). The benchtop pressure drop measured in dynes/cm^2^ is equivalent to the electrical voltage, the benchtop flow rate measured in cm^3^/s is equivalent to the electrical current, the benchtop model material local compliance measured in cm^4^/dynes is equivalent to the electrical capacitance, and the benchtop resistance measured in dynes*s/cm^5^ is equivalent to the electrical resistance. These equivalent electric circuits simulated the coronary tree branches in series/ parallel based on the geometric structure of each patient specific coronary system. Localized resistances along each of the coronary arteries were calculated using Poiseuille’s Law (Eq. 1) derived from vessel radii and length.

1 $$R = \frac{8\mu l }{\pi {r}^{4}}$$

where,

R = resistance (dynes*s/cm^5^)

µ = viscosity of working fluid (3.7 cP)

l = length of vessel (cm)

r = radius of vessel (cm)

**Fig. 3:**
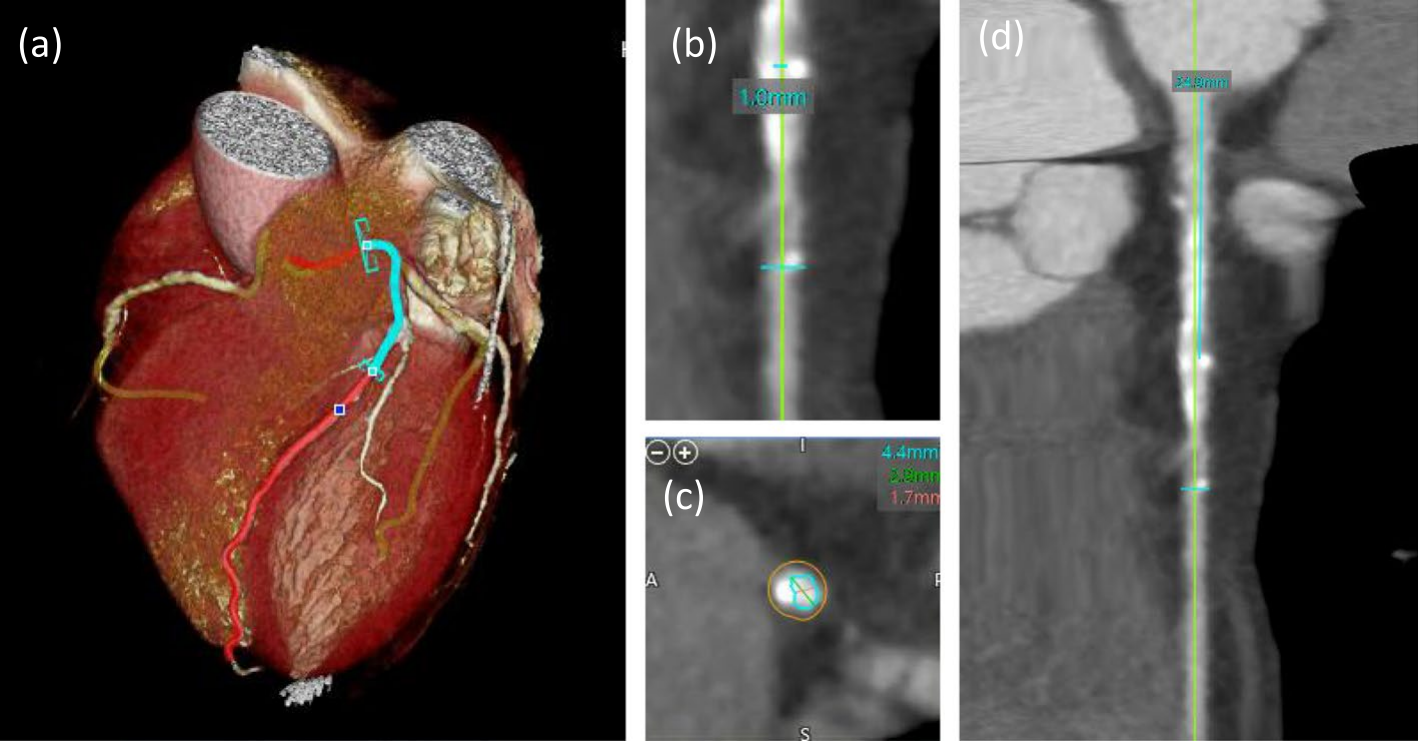
Length and Radii Determination to Calculate Resistance. **a** The three main coronary arteries were automatically segmented from the rest of the heart tissue. The blue highlighted region of the LAD is the location in which plaque buildup is located. **b** The radii of the LAD are measured at the location of the stenosis and is input into Poiseuille’s Law equation to determine the resistance within the vessel at the location of the stenosis. **c** Additional view of stenosis within LAD to determine vessel radius. **d** The length of the vasculature from the ostium to the location of the stenosis is measured and input into Poiseuille’s Law equation to determine the resistance within the vessel at the location of the stenosis

The radii and length of the vasculature were determined within Vital Images cardiac applications by analyzing the multi-planar reconstructions of the CCTA scans (Fig. 3). The radius of the vessels was measured at the location of each stenosis as well as each location that the vessels split into additional branches. The radii measurement was taken as a mean average along the stenosis. The length was determined as the distance from the branch start to the location of the stenosis/next branch split and then added until the distal location of the I-FFR measurement location was reached.

The arterial compliance, or the elasticity and extensibility of the arteries, was combined with the arterial resistance as a parallel circuit and the impedance was calculated across each of the coronary artery branches (Eq. 2). The arterial compliance was determined in a previous publication, Tabaczynski et al. [13, 14], in accordance with the chosen material for the vasculature and was applied to this electrically- equivalent model. The arterial compliance was carried over to the Windkessel, Invasive and Benchtop methodologies creating a uniformity across all cases.

2 $$Z=\frac{1}{\sqrt{{\left(1/R\right)}^{2}+{\left(2\pi C/{T}_{c}\right)}^{2}}}$$

where,

Z = Impedance (dynes*s/cm^5^)

R = Arterial Resistance (dynes*s/cm^5^)

T_c_ = Period of Cardiac Cycle (s)

C = Arterial Compliance (cm^4^/dynes)

For flow management and distal resistance simulation implementation, we designed a three-chamber structure which collected the outflow of the main arteries and daughter branches and was included in our phantom design. The compliance and the resistance of the compliance chamber were attached distally to the vasculature as stated, and so within the electrical circuit it is in series after each of the three main coronary arteries are represented. In Sommer et al., the inherent mean resistance of the chamber was calculated for the LAD, LCX, and RCA, as 1671, 1820, and 591 (dynes*s/cm^5^), respectively. The compliance was calculated as seen in Eq. 3:

3 $${Compliance}_{chamber}=\frac{\Delta V}{\Delta P}= \frac{{V}_{d}-{V}_{s}}{{P}_{s}-{P}_{d}}=\dots = \frac{{P}_{0}{V}_{0}(\frac{1}{{P}_{d}}-\frac{1}{{P}_{s}})}{{P}_{s}- {P}_{d}}$$

where:

V_d_ = Volume during diastole (mm^3^)

V_s_ = Volume during systole (mm^3^)

P_s_ = Pressure during systole (mmHg)

P_d_ = Pressure during systole (mmHg)

P_0_ = Initial Pressure (mmHg) = 760 mmHg/ 1 atm

V_0_ = Initial Volume (mm^3^) = 4.4 × 10^4^ mm^3^

We used a standard total coronary flow rate of 8.33 cc/s when adding up both the left and right side of the coronary tree, the flow rates were added up using parallel/series electrical equations to verify the correct flow rates were being calculated in our models. The distal vascular resistance at different activity states was applied to the model for the LAD, LCX, and RCA at rest, light exercise, and moderate exercise and applied as the most distal resistance in series with each of the three main coronary arteries [12]. Therefore, changes in activity were accounted for in the distal resistance of the three main coronary arteries and not at the specific location of stenosis or proximal to the distal arterioles. The compliance remained a constant during changes in physical activity as well and could be a limitation to the study design. In addition, using the clinical measurements and the resistance derived from Eq. 1, the flow rate at the specific location in which the I-FFR was recorded is then calculated and this process with correlating equations is further described in a later section.

### Flow rate determination

The flow rate was then calculated at the location in which the I-FFR was determined using the electrical equations and resistances calculated from Poiseuille’s Law (Eq. 4).

4 $$I\left(t\right)=\frac{P(t)}{1/\sqrt{(\left[\left(1/R\right)\right]{}^\wedge2+\left[\left(2\pi C/T\_c\right)\right]{}^\wedge2)}}$$

where,

I(t) = Flow rate (cm^3^/s)

P(t) = Pressure (dynes/cm^2^)

R = Resistance (dynes*s/cm^5^)

T_c_ = Period of Cardiac Cycle (s)

C = Compliance (cm^4^/dynes)

The flow rate was determined for the invasive method by using the FFR and the resistances obtained for Poiseuille’s Law. The flow rate was determined for the benchtop method by using the pressure readings recorded during experimentation and the resistances calculated from Poiseuille’s Law.

### Statistical analysis

The Pearson correlation values were calculated to assess the results obtained from the benchtop flow rate (I_B_), Windkessel flow rate (I_W_), and the invasive flow rate (I_I_) at rest, light exercise, and moderate exercise for both the LAD and LCX at the location of the stenosis. Bland Altman plots were created between the Invasive, Electrical and Benchtop flow rates from the average differences of the data.

## Results

We created 5 3D printed patient specific models for this research, including the vasculature, calcification, and a chambered support design. Segmentation of the vasculature and calcification took approximately 1 h and then model design within Autodesk Meshmixer (San Rafael, California) took approximately 2 h. It took approximately 24 h to print one tray of these 5 models on the Stratasys Object 500 multi-material printer (Stratasys, Eden Prairie, MN).

Pressure wave measurements were recorded at flow rates between 80–160 mL/min with varying distal resistance catheters connected to the support chamber to simulate different states of activity for the LAD and LCX for each of the 3D printed models as these were the stenosed vessels. In a previous publication, the correlation between the I-FFR in the angio-suite and the FFR recorded within the 3D printed models at hyperemic conditions presented a mean difference of 0.006 ± 0.118 (average ± 95% CI). Additionally, ROC analysis displayed an area under the curve (AUC) of 0.81 ± 0.065. Therefore, we are able to leverage these 3D phantoms with high accuracy to develop, validate and refine electrically-equivalent theoretical Windkessel models for coronary artery disease hemodynamics studies [20].

Electrically equivalent Windkessel circuits were created of each of the 5-patient specific coronary arterial systems. The resistances were determined along each of the three main coronary arteries and their branches using Poiseuille’s Law on the CCTA scans. Literature review shows that the total input flow rate of the coronary arteries is approximately 8.33 cc/s, therefore we totaled the left and right-side input flow rates to verify that the totaled flow rate was close to that number while accounting for blood flow proceeding out of the aorta as well. Our experiments matched the theoretical simulations as seen in Table 1 as well as verifying that blood flow added up to 8.33 cc/s with appropriate blood volumes flowing through the left coronaries, right coronary and the aorta. As the distal resistance increase, i.e. simulated physical activity decreased, the flow rates through the system decreased proportionally.

**Table 1:** Windkessel input flow rates

Model	Cardiac Location	E2 (cc/s)	E1 (cc/s)	R (cc/s)
**#1**	Left + Right Coronaries	7.64	3.90	2.08
	Leaving Aorta	0.69	4.43	6.25
**#2**	Left + Right Coronaries	7.68	3.91	2.09
	Leaving Aorta	0.65	4.42	6.24
**#3**	Left + Right Coronaries	7.72	3.92	2.09
	Leaving Aorta	0.61	4.41	6.24
**#4**	Left + Right Coronaries	7.70	3.92	2.09
	Leaving Aorta	0.63	4.41	6.24
**#5**	Left + Right Coronaries	7.67	3.91	2.09
	Leaving Aorta	0.66	4.42	6.24

Data is presented as the input flow rate calculated into the coronary tree and exiting the aorta at rest (R), during light exercise (E1), and during moderate exercise (E2). Left and right coronaries indicate the Left Anterior Descending, Left Circumflex, Right Coronary Artery and all of their corresponding branches. ‘Left + Right Coronaries’ and ‘Leaving Aorta’ values when added together is equivalent to 8.33 cc/s, the literature reviewed normal total input flow rate

The local flow rate at the stenosis was then determined at both the LAD and LCX for all 5 patient specific geometries by using the localized resistance at each of these locations determined using electrical equations (Table 2). We hypothesized that the flow rate at the stenosed regions of the vasculature would increase as the activity level increased due to coronary dilation and our hypothesis proved to be significant based on our results. Also, the flow rate at the stenosis strongly correlates with the Windkessel and the Benchtop flow rates calculated as demonstrated by the Pearson coefficients.

**Table 2:** Flow rates at diseased location

Activity State	Cardiac Location	Invasive (cm^3^/s)	Benchtop (cm^3^/s)	Electrical (cm^3^/s)
R	LAD	0.40	0.42	0.52
	LCX	1.14	1.05	1.30
E1	LAD	1.00	1.02	1.29
	LCX	1.14	1.03	1.30
E2	LAD	1.65	1.97	2.49
	LCX	2.26	2.01	2.53

Data is presented as the flow rate calculated at the location of the disease, or the stenosis, during rest (R), during light exercise (E1), and during moderate exercise (E2)

**Fig. 4:**
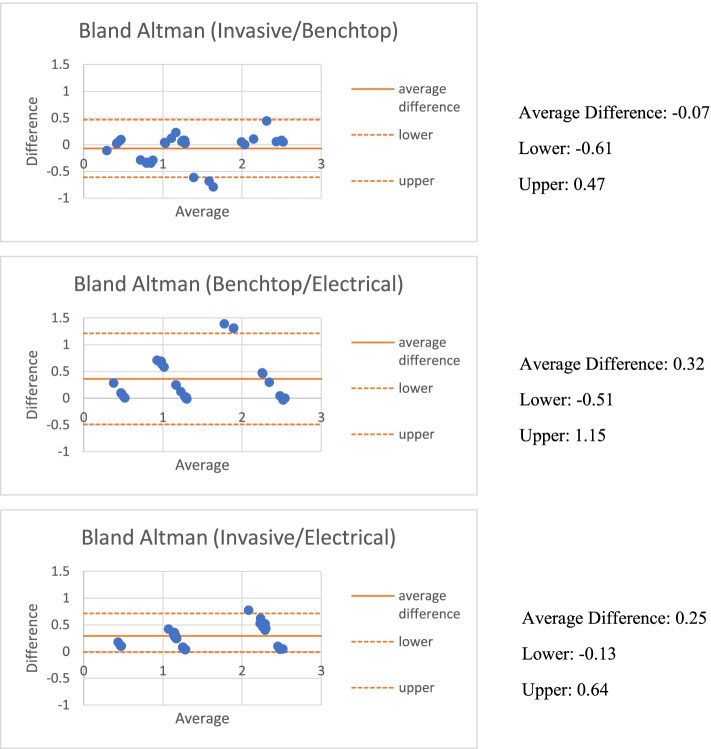
Bland Altman Plot Comparing Flow Rates at Diseased Location. The average difference of the flow rate at the diseased location was determined and compared from the benchtop model, Windkessel electrical model, and angio-suite. Bland Altman plots display these averages and differences

The flow rate was determined at the locations of stenosis for the invasive pressure measurements obtained using the resistances obtained from Poiseuille’s Law on the CCTA scans. The benchtop flow rate, I_I_, and I_W_ all used the resistances obtained along the coronary arteries extrapolated using Poiseuille’s Law from the CCTA scan length and radii measurements. The benchtop flow rate, invasive flow rate, and Windkessel flow rate were then compared using statistical analysis methods including Pearson Correlations between invasive flow rate/ benchtop flow rate, benchtop flow rate /electrical flow rate, and invasive flow rate / electrical flow rate (Table 3). Bland Altman plots demonstrated small disagreements between invasive flow rate / benchtop flow rate, benchtop flow rate / electrical flow rate, and invasive flow rate / electrical flow rate (Fig. 4)_._

**Table 3:** Pearson correlation of flow rates at diseased location

Model	Benchtop/Invasive	Electrical/Invasive	Benchtop/Electrical
#1	1.000	0.984	0.987
#2	0.980	0.981	1.000
#3	0.999	0.982	0.975
#4	0.876	0.998	0.847
#5	0.996	0.981	0.982
Average	0.970	0.981	0.958

## Discussion

This paper investigates the correlation between experimental results in benchtop flow simulations in coronary 3D printed phantoms and a theoretical Windkessel model for the blood circulation where boundary conditions and electrical components physical properties were set to match the experimental flow conditions and physical properties of the 3D printed models. This study displays high levels of correlation between these 3D printed patient specific benchtop models when compared to hemodynamics calculated in electrically-equivalent coronary Windkessel models as well as invasive angio-suite pressure measurements. Theoretical models have a long history of successful use to describe cardiovascular disease [23–28], and in this study, we demonstrated that we can extend that application to characterize the flow in 3D printed models. The results confirm previous findings by Sommer et. al [12] that 3D printed patient specific coronary phantoms can be used to replicate the human arterial anatomy as well as blood flow conditions and extends our understanding and ability to use 3D printing to replicate the distal resistance of the coronary arteries as well as the compliance of the aorta and coronary arteries. Using adjustable distal resistance and compliance, we can create active 3D printed models which replicate the distal resistance of the coronary arteries at varying activity states (rest, light exercise, and moderate exercise) which has a direct impact on the coronary dilation, and, simulate the capillary bed compliance.

Our 3D printed patient specific coronary models offer a unique opportunity to verify the accuracy of the Windkessel electrically equivalent model through the transformation of a fluid dynamic model into an electrical circuit. Using parallel and series equations, we were able to determine the total resistance of each of the three main coronary arteries after determining localized resistances using Poiseuille’s Law on the CCTA patient specific scans. Localized flow rates at the stenosis treated in the angio-suite (with a fractional flow reserve value determined) were then calculated in our electrically equivalent models to predict the flow rate that should be output from our benchtop coronary models. Five patient cases (10 vessels in total) were analyzed to determine the variability between different cases as an effect of lesion characteristics. The changes in flow rate for each case with changes in physical activity state indicated that patient-specific lesion characteristics are hemodynamically significant. This allows us to verify the accuracy of electrically equivalent based calculations by comparing the hemodynamics not only in the angio-suites but also in 3D printed patient specific models. This approach could potentially be used to reverse engineer the 3D printed phantoms after validation. Based on the in-silico simulations, the capacitance and resistance can be adjusted until desired flow dynamics are achieved. Then using the relations between the electrical resistance and the flow resistance, and capacitance and vessel wall compliance, respectively, we can design and select the appropriate 3D printing materials to achieve expected flow conditions.

The input flow rates determined from the Windkessel electrically equivalent model ranged from 2.08 – 7.72 cc/s displaying that changes in physical activity has great impact on the diseased vessel hemodynamics. The intensity of the blood pumping or the flow rate varies considerably in regards to the metabolic rate of the body based on activity level ranging from at rest to maximum activity level. The diameter of the coronary arteries directly relates to the flow rate that it is destined to supply and that can be seen in what is known as “cube’s law.” This phenomenon proposes that the flow rate is proportional to the cube of the vessel diameter. Therefore, this change in input flow rate is quite evident with a change in physical activity due to vessel dilation.

There are a few limitations related to the phantom design process. Five CCTA volumes were used with reduced motion artifacts and calcium burden to alleviate potential segmentation limitations; however, error still may have occurred as vessel diameters are between 3- and 5 mm- with significant flow passing through the vessels. In regards to detection of soft and hard plaques, the segmentation process may introduce error specifically with the soft plaques that hold a lower Hounsfield unit difference from the vasculature. Cross validation was used to reduce this error by having 2 separate users segment the vasculature and segmentations were compared. During the process of vessel sculpting and artifact elimination within Meshmixer, the manipulation of single triangular vertices has potential to affect the accuracy of the overall vessel geometry. Upon importation of the support chamber, tubing was added to the distal end of the coronary arteries to allow for vasculature to pass through the chamber perpendicularly. However, this process of tubing appendage may affect the tortuosity of the distal coronary arteries. Another potential limiting factor of the benchtop design is that the elasticity of the vessel material may deform over time and potentially affect the hemodynamics of the benchtop testing performed. To reduce this, we built the 5 models and tested them as new models that had never been previously tested. In regards to our benchtop design, the catheters attached to the distal ends of the coronary arteries were created to output resistances in increments of 10,000 dynes*s/cm^5^ and therefore induced a small error percentage to our system [12, 22].

There are also a few potential limitations in regards to the Windkessel electrically equivalent model design. In order to verify that the input flow rates within the left and right side of the coronary tree were correct, it was assumed based on extensive literature review that an input flow rate of 8.33 cc/s within the coronary tree is normal. Therefore, the addition of all coronary branches for each model was compared to this value to verify accuracy of our electrical model. Additionally, an input voltage (pressure) was assumed to be 100 mmHg for our electrical equations which is within the physiologically accurate range but could have varied within each model. Another limitation to the Windkessel electrically equivalent model design is that we used Poiseuille’s Law to derive the resistance. Poiseuille’s Law assumes laminar flow through a rigid tube of constant circular cross section and the coronary arteries neither output laminar flow nor are they rigid. Therefore, this assumption may reduce the accuracy of the modeling. A model of fluid flow through an elastic tube with variable radii would need to be applied to the coronary vasculature for increased accuracy. With that being said, when we evaluated the flow rates at resting and hyperemic conditions in Table 2, the ratio of these flow rates was not equivalent to the invasive fractional flow reserve. This proves that there is an introduction of error from most likely the calculated resistances using Poiseuille’s Law.

## Conclusions

We developed 3D printed patient specific benchtop phantoms and leveraged these 3D phantoms to develop, validate and refine electrically-equivalent theoretical Windkessel models for coronary artery disease hemodynamics studies. These 3D printed models have the capability to simulate the distal arterial resistance as well as the compliance of the coronary tree while maintaining geometric accuracy with the addition of hard plaques. Our results present an innovative means to test the accuracy of theoretical models by determining the localized resistances and flow rates across the stenosis creating turbulent flow. This allows for a better understanding and verification of the hemodynamic outputs obtained from our 3D printed benchtop models and input into electrically equivalent theoretical models.

## Data Availability

The datasets used and/or analyzed during the current study are available from the corresponding author on reasonable request.

## Abbreviations

CCTA: Coronary Computed Tomography Angiography
LAD: Left Anterior Descending
LCX: Left Circumflex
RCA: Right Coronary Artery
STL: Stereolithographic
